# Anti-Mullerian Hormone and conception timing as predictors of live births in cancer patients using fertility preservation: a systematic review

**DOI:** 10.3389/fonc.2025.1683794

**Published:** 2025-10-09

**Authors:** Hillary Klonoff-Cohen, Mounika Polavarapu, Neha Ramachandran

**Affiliations:** ^1^ Department of Family Medicine and Public Health, University of California, San Diego, San Diego, CA, United States; ^2^ Department of Population Health, University of Toledo, Toledo, OH, United States; ^3^ Evidence-Based Practice Research Program, Mayo Clinic, Rochester, MN, United States

**Keywords:** Anti-Mullerian Hormone, neoplasm, cancer, fertility preservation, pregnancy

## Abstract

**Objectives:**

Among women of childbearing potential aged 15–39, cancer incidence is 52.3 per 100,000 annually. Women newly diagnosed with cancer often have just 2–6 weeks to decide whether to pursue fertility preservation (FP) before commencing treatment. The recommended waiting period to conceive post-treatment ranges from 6 months to 5 years, depending on cancer type, treatment, and age. With >18 million young adult cancer survivors worldwide, identifying factors affecting fertility preservation and live birth outcomes is more critical than ever. This is the first systematic review to explore whether AMH levels before, during, or after chemotherapy predict pregnancy outcomes resulting from re-utilization of stored oocytes/embryos or spontaneous conception in cancer patients undergoing FP. It also evaluates the optimal timing for post-treatment AMH recovery and how this may inform fertility success and decision-making for cancer patients pursuing FP.

**Methods:**

A review of PubMed and Web of Science identified 458 studies until November 2024. After a full-text review of 38 studies, seven met the eligibility criteria: if they were peer-reviewed, in English, enrolled female cancer patients undergoing FP before chemotherapy, measured AMH, and reported pregnancy or live birth rates after chemotherapy. Study quality and relevance were categorized as high, moderate, or low. Of the seven studies, one was highly relevant, four were moderately relevant, and two were of lower relevance.

**Results:**

The majority of studies focused on patients with breast cancer or lymphoma, comprising three prospective and four retrospective designs. Oocyte cryopreservation emerged as the most commonly used fertility preservation method. Among those who used stored specimens, baseline AMH levels ranging from ~2.1 to 2.8 ng/mL were related to live birth rates of 35–42%. Notably, spontaneous conception was more frequent than assisted reproduction. AMH recovery timelines varied widely, with follow-up periods spanning 1 to 36 months, yet no clear optimal timeframe for ovarian reserve restoration emerged.

**Conclusion:**

In female cancer patients, pre-treatment Anti-Müllerian Hormone (AMH) levels may offer valuable insight to help inform fertility preservation decisions aimed at achieving future live births. This first-of-its-kind systematic review lays the groundwork for future research by identifying key knowledge gaps and emerging areas of clinical relevance.

## 1 Introduction

Preserving fertility and the constraints of a limited reproductive window are key concerns for many cancer survivors (1–3). For young survivors, achieving remission and remaining cancer-free are paramount, but for many, the ability to start a family represents a crucial milestone in reclaiming a sense of normalcy (4–6). However, delaying cancer treatment to pursue fertility preservation can have serious consequences for prognosis. A recent study found that postponing treatment by just one month, regardless of the treatment modalities (i.e., surgery, chemotherapy, and radiation), was associated with a 6-13% increase in mortality risk, with the danger compounding as delays lengthen (7).

Anti-Müllerian Hormone (AMH) is a widely recognized marker of ovarian reserve and has been proposed as a valuable tool in fertility counseling for cancer patients considering fertility preservation before undergoing gonadotoxic chemotherapy. However, the impact of AMH fluctuations on key clinical outcomes, such as pregnancy rates and live birth success resulting from re-utilized oocytes/embryos or spontaneous conception, remain largely unknown (1, 8–11). Addressing these gaps is essential for improving fertility counseling and optimizing reproductive decision-making for cancer survivors.

### 1.1 AMH and fertility preservation

For cancer survivors considering fertility preservation, pre-treatment AMH may provide an estimate of ovarian reserve, aiding in counselling and optimizing fertility preservation strategies.

For those undergoing fertility preservation, post-chemotherapy AMH recovery levels may provide valuable insights into the recovery of follicle growth in the functional ovarian reserve (12, 13). Sustained low AMH concentrations post-chemotherapy, especially with alkylating agents, may significantly decrease the conception window, increasing the risk of premature ovarian failure and decreasing the likelihood of pregnancy in a cancer patient population (13).

The decision to delay cancer treatment in order to pursue fertility preservation through oocyte or embryo cryopreservation emphasizes the need to better understand the potential utility of AMH as a biomarker for reproductive outcomes. While the current evidence base is limited, AMH may offer insight into chemotherapy-induced ovarian dysfunction (14–17) and could potentially inform the likelihood of future clinical pregnancy and live birth using stored oocytes/embryos from fertility preservation. Further research is needed to clarify AMH’s prognostic value and to determine its role in guiding fertility preservation counseling and decision-making in the oncofertility setting.

At present, no specific guidelines exist to help couples assess whether their chances of achieving a healthy live birth are better with fertility preservation techniques compared to relying solely on natural conception after cancer treatment.

### 1.2 Study objectives

AMH’s ability to predict spontaneous pregnancy has been assessed across various populations, including healthy but predominantly obese women aged 30-44, cancer patients treated with GnRH, and young breast cancer patients not using fertility preservation (18–21). Thus far, the literature has only extrapolated the role of AMH to cancer patients undergoing fertility preservation without accompanying evidence-based knowledge.

Our study aims to address a very specialized gap by conducting a systematic review of the existing literature to evaluate whether AMH levels prior to, during, or post-chemotherapy, could serve as a reliable predictor of success rates (including re-utilization of oocytes/embryos or unassisted spontaneous conception) for pregnancy and healthy live births among cancer patients undergoing fertility preservation The absence of research evaluating predictive biomarkers of fertility preservation success, particularly the likelihood of achieving a healthy pregnancy or live birth after chemotherapy, underscores the need for further investigation in this field.

The second aim of this systematic review is to identify an optimal time interval (i.e., a suitable period to get pregnant) after chemotherapy when AMH levels tend to recover most robustly among those women undergoing fertility preservation.

## 2 Methods

### 2.1 Search strategy

Two authors (NR and HKC) independently conducted a systematic search following PRISMA guidelines. The search included PubMed and Web of Science to identify relevant studies published up to November 2024. After removing duplicates, studies were initially screened based on their titles and abstracts, with full texts retrieved for the remaining studies. Two authors (HKC and MP) then evaluated the full texts against the predefined inclusion and exclusion criteria to finalize the studies for the systematic review.

### 2.2 Inclusion exclusion criteria

Studies were included if they: (i) were peer-reviewed, original research articles published in English, (ii) enrolled female cancer patients who underwent fertility preservation (e.g., oocyte or embryo cryopreservation) prior to cancer treatment, (iii) measured anti-Müllerian hormone (AMH) as one of the biomarkers, and (iv) reported pregnancy and/or live birth rates after chemotherapy as primary or secondary outcomes.

Studies were excluded if: (i) they did not involve fertility preservation prior to cancer treatment or did not evaluate subsequent IVF outcomes, (ii) they did not measure AMH, (iii) if the study sample consisted of childhood cancer patients who exclusively used tissue cryopreservation (iv) they were not in English or included only animal or laboratory-based research, male, transgender, or pediatric populations, or (v) they were reviews, systematic reviews, meta-analyses, case reports, conference abstracts, committee announcements, or protocol papers without original data.

### 2.3 Data extraction

The authors collectively extracted and compiled data from the selected studies, ensuring agreement, consistency, and accuracy. The extracted data included study details such as the first author, year of publication, and country, as well as study characteristics, including the hypothesis, study design, cancer type, sample size, and participant age. Additionally, data were collected on fertility preservation, AMH measurement techniques, the interpretation of AMH levels, and reported pregnancy outcomes. Specific details on AMH measurement techniques included the timing of measurements, AMH levels, units of measurement, and the type of values were reported.

### 2.4 Study quality and relevance assessment

To evaluate the relevance and quality of included studies in addressing our research hypothesis, a scoring system was developed, reflecting the overall quality and rigor of the studies as well as their alignment with our specific research question (**Table 1**).

**Table 1:** Study quality assessment criteria based on relevance to our hypothesis.

Criteria	Scoring categories	Significance
Study Design	1 - Retrospective study 2 - Prospective study	Prospective studies provide stronger evidence due to controlled follow-up.
Type of Cancer Studied	1 - Mixed cancer types 2 - Majority with one cancer type 3 - Focused on a single cancer type	AMH and fertility outcomes may vary by cancer type and type of treatment
Sample Size	1 - Fewer than 50 participants 2–50 to 200 participants 3 - More than 200 participants	Larger sample sizes increase statistical power and reliability of findings.
Timing of AMH Measurement	1 - Baseline only 2 - Measured up to 1 year 3 - Measured up to 2 years 4 - Measured up to 3 years	Longer follow-up provides better insights into changes in ovarian reserve post-treatment.
AMH Measurement Technique	1 - Not reported 2 - ELISA technique	AMH measurement accuracy affects its reliability in fertility assessments.
Reporting on Participant Age	1 - Reported at a single time point 2 - Reported separately for fertility preservation and later use	There are age-related changes in AMH levels and fertility success.
Pregnancy Outcome Reporting	1 - No pregnancy or live birth reported 2 - Pregnancy reported 3 - Live birth reported	Pregnancy and live birth outcomes indicate the success of fertility preservation.
Use of stored specimens from fertility preservation vs. natural conception	1 - Unknown 2 - Natural conception 3 - Used stored specimens from fertility preservation	Differentiating the importance of fertility preservation vs natural conception for cancer patients.
Total possible score	22	

Two reviewers (HKC, MP) assigned a score for each study ranging from 7 to 20, based on factors including study design, type of cancer, sample size, technique and timing of AMH measurement, patient age at specimen use for fertility preservation, and presence of pregnancy outcomes (**Table 1**). Given the uniqueness of the hypothesis and paucity of availability studies, relevance was determined by the alignment with our study hypothesis. Studies scoring above 15 were classified as high-quality and highly relevant, those between 11 and 14 as moderate-quality and moderately relevant, and those between 7 and 10 as lower-quality and less relevant.

## 3 Results

### 3.1 Study selection

**Figure 1:**
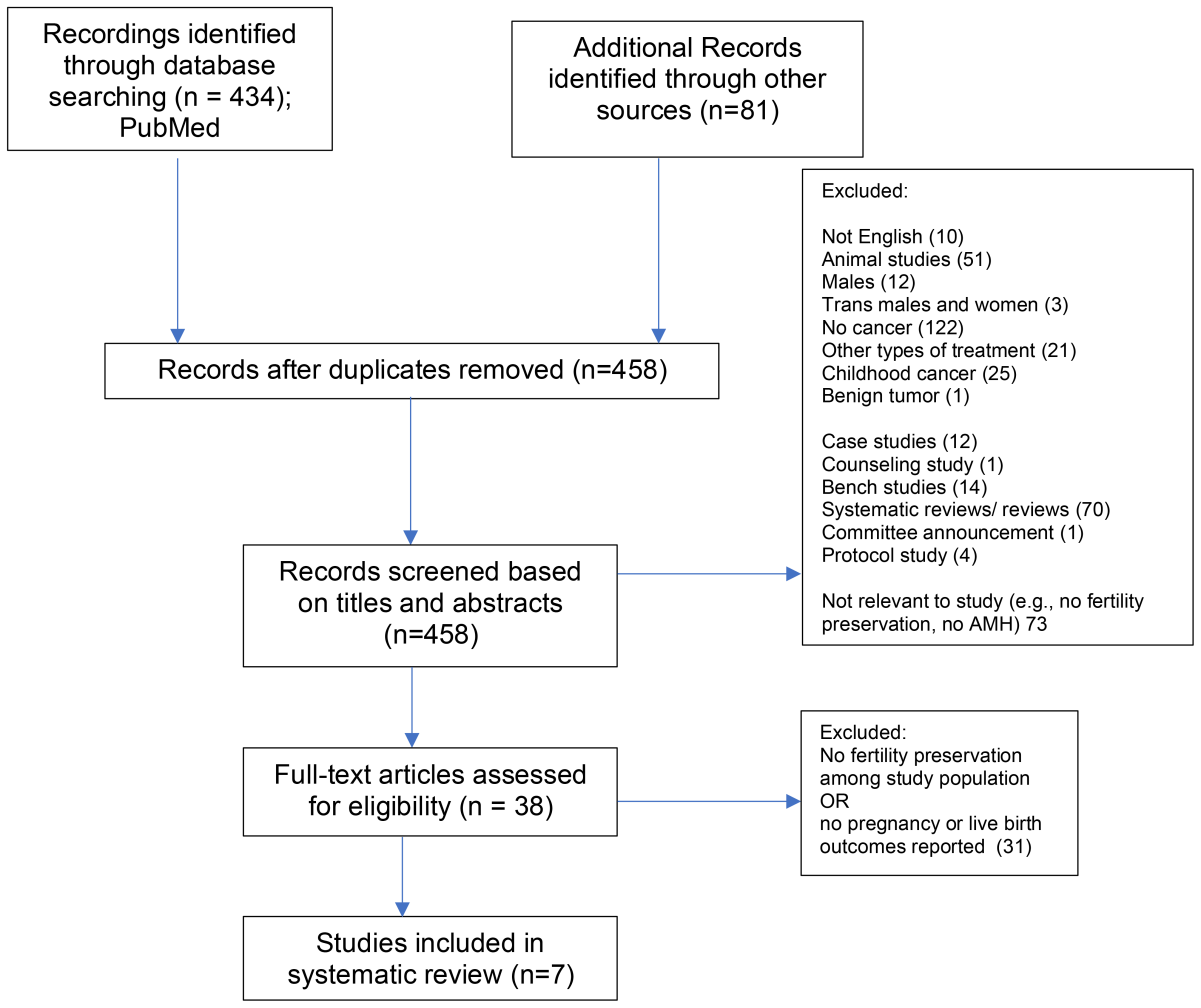
PRISMA Chart. *From*: Page MJ, McKenzie JE, Bossuyt PM, Boutron I, Hoffmann TC, Mulrow CD, et al. The PRISMA 2020 statement: an updated guideline for reporting systematic reviews. BMJ 2021;372:n71. doi: 10.1136/bmj.n71

The process of study identification and selection, based on PRISMA guidelines, is illustrated in **Figure 1**. The initial search strategy identified 525 studies. After removing duplicates, 458 articles remained and were screened based on their titles and abstracts. From this, 38 articles were shortlisted for full-text review and assessed against the inclusion criteria.

The most frequent reasons for exclusion during the full-text review included a lack of cancer diagnosis, the absence of fertility preservation or Anti-Müllerian Hormone (AMH) assessments, and articles that were systematic or review papers. Other common grounds for exclusion involved non-English publications, animal studies, and populations outside the review scope (e.g., exclusively male, transgender-inclusive, or childhood cancer cohorts). Additionally, an attempt was made to identify relevant studies through reference lists, but no additional studies were found.

### 3.2 Study characteristics

Ultimately, seven studies met the inclusion criteria and were included in the review (**Table 2**) (22–28). These studies, published between 2016 and 2023, examined fertility preservation among cancer patients and highlighted AMH as a key biomarker of ovarian reserve. Of these, three were prospective (22–24) and four were retrospective (25–28). These studies took place in Portugal (22), Spain (25), Italy (23, 24, 28), and the United States (26, 27). Sample sizes ranged widely from fewer than 50 participants in smaller cohorts (e.g., Silva et al. (22)) to larger-scale analyses exceeding 5000 (e.g., Cobo et al. (25)).

**Table 2:** Characteristics of included studies.

Author, year, and country	Hypothesis	Study design	Type of cancer	Sample size and age	Fertility preservation (Yes/No)	Interpretation of AMH levels	Information about pregnancy outcomes
Ciccarone et al., 2020: Italy (23)	To describe a population of patients referred for fertility preservation (FP), how to efficiently provide FP care	Prospective	Hodgkins Lymphoma; Non-Hodgkins Lymphoma, Breast Cancer, Leukemia, Other solid/hematological cancers	A total of 251 cancer patients with median age of 31 years (3–44 years)	Among 251 cancer patients, 44 did not undergo FP, 135 received GnRHa, 31 had GnRHa + oocyte cryopreservation, and 41 had GnRHa + ovarian tissue cryopreservation	At follow-up (1, 6, 12, 24, and 36 months) after chemotherapy completion a statistically significant decrease in AMH levels was observed.	Seven patients had post- cancer treatment pregnancies all of which occurred spontaneously. FP offered to everyone. Incidence rate of pregnancies and live birth deliveries from FP is still not available since follow-up is not long enough (need 3–5 yrs).There were 6 live births and one first-trimester miscarriage within the three FP groups. It was not broken down by type of FP.
Ciccarone et al., 2023: Italy (24)	To assess Hodgkin’s lymphoma patients involved in fertility preservation counseling and analyze the impact of ABVD on ovarian function	Prospective cohort	Hodgkins Lymphoma	A total of 270 cancer patients with median age of 28 years (18–40 years)	Among 270 cancer patients, 32 did not undergo FP, 135 received GnRHa, 73 had GnRHa + oocyte cryopreservation, and 65 had GnRHa + ovarian tissue cryopreservation. No information on patients returning to use FP specimens	Average AMH level showed a statistically significant reduction at 6 months post-chemotherapy (p=0.05). At 12 months, AMH returned to near pre-chemotherapy values.	Nine post-chemotherapy pregnancies in the study group (FP and no FP), all spontaneous with seven natural deliveries and one cesarean. All were healthy babies. One pregnancy ended in an elective abortion.
Cobo et al., 2018: Spain (25)	To determine whether the indication for fertility preservation (FP) is related to success in IVF cycles after elective-FP (EFP) for age-related fertility decline and FP before cancer treatment (Onco-FP	Retrospective cohort	Breast cancer (64.6%), Hodgkin’s lymphoma (11.6%), non-Hodgkin’s lymphoma (5.2%),and other malignancies (18.6%)	The Onco-FP group (n=1073) had a mean age of 32.3 years, while the Elective FP group (n=5289) had a mean age of 37.2 years	Among cancer patients, 1073 underwent oocyte cryopreservation, of whom 80 underwent fresh embryo transfer and 21 underwent cryotransfer of surplus embryos	Despite AMH levels being higher in the Onco-FP group, there was no statistical difference between groups	Patients under 36 years of age had a significantly higher cumulative probability of live birth compared to those over 36 in elective fertility preservation (EFP) (P < 0.0001), with better outcomes when more oocytes were available for IVF. Clinical pregnancy rates were 41.4% for fresh embryo transfer (ET) cancer patients and 32.1% for frozen ET cancer patients. The cumulative live birth rate was 35.2% in the onco-fertility preservation (FP) group and 33.9% in the elective FP group, with no significant difference. For fresh embryo transfers, elective FP resulted in 115 live births (12%), while onco-FP had 18 live births (7.4%). In the cryopreserved surplus group, elective FP led to 47 live births (24.8%), whereas onco-FP had 7 live births (56.9%).
Dolinko et al., 2018: USA (27)	To evaluate whether there is an association between any cancer and/or type of cancer, and response to ovarian stimulation for egg and embryo banking	Retrospective cohort	Multiple cancers	No cancer group (n=664) with mean age 34.6 years; Local cancer (n= 105) with mean age 33.6 years; Systemic cancer (n=42) with mean age 27.1 years	Among 147 patients undergoing fertility preservation, 131 did not return, 15 returned to use frozen embryos, and 1 returned to use frozen oocyte	Women with systemic cancer had lower baseline AMH levels than women with local cancer or no cancer.	15 patients returned to use frozen embryos for a total of 18 transfers. Live birth rates were 40% per cycle or 42.1% per ET and 50% per women treated. Frozen ET deliveries resulted in 7 singleton births and 1 resulted in a set of twins. Birth weights ranged from 2381–4706 g. one women returned to use frozen oocytes, but she did not get pregnant.
Ferro et al., 2023: Portugal (28)	To evaluate the impact of early treatment on ovarian reserve and observe fertility preservation outcomes	Retrospective cohort	Hematological malignancies	A total of 61 cancer patients with mean age of 25.97 years (15–36 years)	Among cancer patients: 26 did not undergo FP, 18 had oocyte cryopreservation, 14 had ovarian tissue cryopreservation, 2 had simultaneous embryo + oocyte cryopreservation, and 1 had embryo cryopreservation. Four returned to use cryopreserved material.	Cancer treatments caused a decrease in ovarian reserve, specifically, a decrease in AMH.	Moreover, pregnancy after treatment occurred in 11 cases and 10 of them were spontaneous. Among the 4 women who resorted to cryopreserved material (1 for each cryopreservation technique), only 1 became pregnant, using the cryopreserved oocytes. Most pregnancies occurred at least 2 years after diagnosis.
Nurudeen et al., 2016: USA (26)	To evaluate fertility preservation decisions and compare controlled ovarian stimulation (COS) and assisted reproductive technology (ART) outcomes between newly diagnosed cancer patients and age-matched healthy controls	Retrospective	Majority with breast cancer and remaining with hematologic cancers	A total of 82 cancer patients with mean age of 33.6 years (21–44 years)	A total of 49 cancer patients underwent oocyte (n=11) or embryo (n=37) cryopreservation. Two patients returned to use cryopreserved embryos.	Baseline anti-Mullerian hormone levels and body mass indices were similar among fertility preservation patients and controls.	One of the two returning patients delivered a healthy baby, and the other was still pregnant by the end of the study
Silva et al., 2019: Portugal (22)	To measure levels of ovarian reserve in a cohort of young women with breast cancer exposed to chemotherapy to identify adverse reproductive health outcomes	Prospective cohort	Breast cancer	A total of 46 cancer patients with median age of 33 years (25–39 years)	Among cancer patients, 29 underwent FP: 25 oocyte, 2 embryo, 2 ovarian tissue cryopreservation. None attempted to get pregnant during the study time period	At the last follow up, 35 patients had AMH below the expected values for age. Age and baseline AMH were positively correlated with AMH at the last follow-up. AMH levels were higher in the group of patients treated with trastuzumab and lower in those under hormonal therapy, at the last follow-up.	4 pregnant (among which group is unknown)

Across the included studies, the most frequent diagnoses were breast cancer (22, 25–27) and Hodgkin’s or non-Hodgkin’s lymphoma (23, 24, 28), with some also including patients with other hematologic malignancies. While several studies exclusively enrolled cancer patients, subdividing them according to the treatments received (22–24, 28), others incorporated comparison groups, such as age-matched healthy individuals (26), elective fertility preservation cohorts (25), or those with male-factor infertility (27).

AMH levels were measured at baseline before chemotherapy in all included studies, with several also reporting repeated assessments during or after treatment to evaluate changes in ovarian reserve (22–28).

### 3.3 Quality and relevance assessment of included studies

The quality and relevance assessment of the included studies are presented in **Table 3**. Among the seven studies evaluated, one study (24) scored 16, classifying it as highly relevant (score >15). Four studies (22, 23, 25, 28) scored 13 or 14, placing them in the moderate relevance category (scores between 11 and 14). Finally, Nurudeen et al., scored 9, and Dolinko et al., scored 10, classifying them as lower relevance (scores between 7 and 10) (26, 27).

**Table 3:** Study quality assessment based on relevance to our hypothesis.

Author	Study design	Type of cancer	Sample size	Timing of AMH	AMH measurement technique	Reporting on participant age	Pregnancy outcome	Total score
Ciccarone et al., 2020 (23)	1	1	3	4	1	1	3	14
Ciccarone et al., 2023 (24)	2	3	3	2	2	1	3	16
Cobo et al., 2018 (25)	1	2	3	1	1	2	3	13
Dolinko et al., 2018 (27)	1	1	2	1	1	1	3	10
Ferro et al., 2023 (28)	1	3	2	3	2	1	1	13
Nurudeen et al., 2016 (26)	1	2	2	1	1	1	1	9
Silva et al., 2019 (22)	2	3	1	2	2	1	2	13

### 3.4 Cancer patients undergoing fertility preservation

The studies by Dolinko et al. (27), Ciccarone et al. (2020) (23), Nurudeen et al. (26), and Cobo et al. (25) included only cancer patients who had undergone fertility preservation. In contrast, other studies included mixed populations: Ciccarone (2023) (24) reported that 88% of participants had used fertility preservation, Ferro reported 58% (28), and Silva reported 76% (22).

### 3.5 AMH levels

All extracted data on AMH levels, including timing of AMH measurements, AMH levels, and AMH measurement assay techniques, are summarized in **Table 4**.

**Table 4:** Summary of AMH levels in cancer patients undergoing fertility preservation and control groups.

Author and year	Timing of AMH measurement	AMH levels	Unit	Type of reported values	AMH measurement technique
Ciccarone et al., 2020 (23)	Baseline AMH among cancer patients	1.7 (0.0-16.0)	ng/ml	median and range	Not reported
	T1 (1 month after chemotherapy)	0.1 (0–5)	ng/ml	median and range	
	T6 (6 months after chemotherapy)	0.1 (0-2.8)	ng/ml	median and range	
	T12 (12 months after chemotherapy)	1.21 (0-5.8)	ng/ml	median and range	
	T24 (24 months after chemotherapy)	0.92 (0-3.7)	ng/ml	median and range	
	T36 (36 months after chemotherapy)	1.5 (0.02-1.9)	ng/ml	median and range	
Ciccarone et al., 2023 (24)	Baseline AMH among cancer patients	1.69 (0.0-15.9)	ng/ml	median and range	ELISA
	AMH at 6 months after chemotherapy	0.56 (0.0-3.9)	ng/ml	median and range	
	AMH at 12 months after chemotherapy	1.6 (0.0-5.8)	ng/ml	median and range	
Cobo et al., 2018 (25)	Baseline AMH among patients undergoing elective FP	10.9 +/-11.2	pmolo/l	mean +/- SD	Not reported
	Baseline AMH among cancer patients using FP	15.4 +/-17.2	pmolo/l	mean +/- SD	
Dolinko et al., 2018 (27)	Baseline AMH in the No cancer group	3.4 ± 3.3	ng/ml	mean +/- SD	Not Reported
	Baseline AMH in the Local cancer group	2.8 ± 2.7	ng/ml	mean +/- SD	
	Baseline AMH in the Systemic cancer group	2.0 ± 2.2	ng/ml	mean +/- SD	
Ferro et al., 2023 (28)	Baseline AMH Overall age group	2.19 +/- 1.89 (0.01-3.70)	ng/ml	mean +/- SD (95% CI)	Roche Beckman Coulter Kit
	Post Chemo AMH (around 2 years) Overall age group	0.52 +/- 0.06 (0.06-7.70)	ng/ml	mean +/- SD (95% CI)	
	Baseline AMH (25–30 years old)	2.279 +/- 1.775 (0.250-4.900)	ng/ml	mean +/- SD (95% CI)	
	Post Chemo AMH (around 2 years) (25–30 years old)	0.479 +/- 0.811 (0.010-0.510)	ng/ml	mean +/- SD (95% CI)	
Nurudeen et al., 2016 (26)	Baseline AMH among cancer patients	1.3 (0.5, 3.7)	ng/ml	median and IQR	Not reported
	Baseline AMH among controls	0.8 (0.4, 2.4)	ng/ml	median and IQR	
Silva et al., 2019 (22)	Baseline AMH	3.07 +/-2.95	ng/ml	mean +/- SD	Ultrasensitive AMH ELISA assay kit (Ansh Lab)
	AMH During Chemo	0.30+/-0.50	ng/ml	mean +/- SD	
	AMH 1 month after chemo	0.15 +/-0.46	ng/ml	mean +/- SD	
	AMH Last available follow-up	0.32 +/-0.68	ng/ml	mean +/- SD	

#### 3.5.1 Baseline AMH

At baseline, AMH levels were highest in healthy controls (Dolinko et al., 2018: 3.4 ± 3.3 ng/ml) (27) and in breast cancer patients before chemotherapy (Silva et al., 2019: 3.07 ± 2.95 ng/ml) (22). Compared to breast cancer patients, those with hematologic malignancies had lower baseline AMH levels, as reported by Ferro et al., 2023: 2.19 ± 1.89 ng/ml (28); Ciccarone et al., 2020: 1.7 ng/ml (0.0-16.0 range) (23); and Ciccarone et al., 2023: 1.69 (0.0-16.0) (24).

Similarly, in a cohort of predominantly newly diagnosed breast and some hematologic cancer patients, Nurudeen et al. observed a median baseline AMH level of 1.3 ng/ml (IQR: 0.5 - 3.7) (26). Among cancer patients undergoing fertility preservation, Cobo et al. included primarily breast cancer patients, reporting baseline AMH levels of 15.4 ± 17.2 pmol/l (25), which converts to 2.16 ± 2.41 ng/ml using the standardized conversion factor (1 pmol/l ≈ 0.14 ng/ml). This value is comparable to other studies.

AMH patterns may, in part, reflect the younger ages of the study sample. For example, the mean or median ages at baseline were 33.6 years (range 21-44) in Nurudeen et al. (26), 25.97 years (range 15-36) in Ferro et al. (28), 28 years (range 18-40) in Ciccarone et al., 2023 (24), and 32.3 years at baseline Cobo et al. (25).

#### 3.5.2 AMH during chemotherapy

Among the studies included in this review, Silva et al. was the only one to measure AMH levels during chemotherapy (22). In breast cancer patients, AMH levels dropped from 3.07 ng/ml at baseline to 0.30 ± 0.50 ng/ml during chemotherapy, highlighting the immediate gonadotoxic effects of cancer treatment (22).

#### 3.5.3 AMH after chemotherapy

Across all studies, AMH levels remained low after chemotherapy, with varying degrees of recovery depending on cancer type, treatment regimen, and patient age.

In breast cancer patients, Silva et al. reported significantly reduced mean AMH levels post-treatment, dropping from 3.07 ng/ml at baseline to 0.15 ± 0.46 ng/ml one month after chemotherapy (22). At the last available follow-up, AMH levels showed only minimal improvement to 0.32 ± 0.68 ng/ml, indicating limited ovarian function recovery.

For patients with hematologic malignancies, long-term recovery of AMH was similarly limited among those undergoing fertility preservation or using natural conception. Ferro et al. observed a significant decline from 2.19 ± 1.89 ng/mL at baseline to 0.52 ± 0.06 ng/mL two years after chemotherapy, highlighting a sustained negative impact on ovarian reserve (28).

A longitudinal assessment by Ciccarone et al., 2020 (23), which included patients with Hodgkin’s lymphoma, non-Hodgkin’s lymphoma, breast cancer, and leukemia, found AMH levels fluctuated over time rather than a linear recovery trajectory. However, median AMH levels remained below baseline throughout the follow-up.

The degree of AMH reduction varied by treatment regimen. BEACOPP (bleomycin, etoposide, doxorubicin, cyclophosphamide, vincristine, procarbazine, prednisolone) resulted in the most substantial decline (28), whereas ABV-treated Hodgkins’ lymphoma patients exhibited partial AMH recovery (23, 28). Ciccarone et al., 2023 reported that in the entire ABVD cohort, median AMH initially declined but rebounded, approaching slightly below baseline levels (24). Similarly, Ferro et al. found that AMH levels in ABVD-treated patients were significantly higher than those receiving other regimens (1.573 ± 1.385 [0.060–3.700] vs. 0.342 ± 0.672 [0.010–3.00] ng/mL, p = 0.058), though still lower than baseline values (28).

The significant decline in AMH levels was particularly pronounced in younger patients (ages 25–30), whose AMH levels dropped markedly from 2.279 ± 1.775 ng/mL before treatment to 0.479 ± 0.811 ng/mL afterward (p = 0.033) (28); however, they did not compare this drop in older patients The pronounced drop in AMH was likely due to the BEACOPP chemotherapy regimen which is very gonadotoxic.

#### 3.5.4 Timing of AMH measurements after chemotherapy among fertility preservation patients

The second aim of this systematic review was to identify an optimal time interval after chemotherapy when AMH levels would tend to recover most robustly.

Ciccarone et al., 2023 reported that in hematological patients, median AMH levels initially declined to 0.56 ng/ml (0.0-3.9) at six months post-chemotherapy but rebounded to 1.6 ng/ml (0.0-5.8) by 12 months, approaching but remaining slightly below the baseline of 1.69 ng/ml (0.0-15.9) (24).

Ciccarone et al., 2020 assessed AMH recovery at 1, 6, 12, 24, and 36 months post-chemotherapy (23). After an initial improvement within the first year, median AMH levels dropped at 24 months before slightly rebounding at 36 months. This pattern could potentially be attributed to the very small sample sizes during follow-up (T0: n=219; T1: n=64; T6: n=27; T12: n=21; T24: n=18; T36: n=9).

Silva et al. followed premenopausal breast cancer patients for 6–35 months post-chemotherapy, and AMH levels remained significantly reduced at the last available follow-up compared to baseline (22).

The reviewed studies did not establish a uniform timeframe for ovarian reserve restoration. Follow-up periods varied markedly across studies, with 2 years reported by Ferro et al. (28), 36 months by Ciccarone et al., 2020 (23), 12 months by Ciccarone et al., 2023 (24), and individualized follow-up schedules by Silva et al. (22), thereby preventing a clear consensus on the optimal timeframe for AMH recovery.

### 3.6 Fertility preservation

Among the seven studies, oocyte cryopreservation emerged as the most frequently utilized fertility preservation method (22, 24, 25). Cobo et al. reported the largest cohort, with 1,073 patients undergoing oocyte cryopreservation (25).

Embryo cryopreservation was less commonly used, with some patients opting exclusively for this method (26, 28).

Ovarian tissue cryopreservation was more frequently employed in patients receiving highly gonadotoxic chemotherapy (23, 24, 28), and was often combined with GnRHa therapy to further protect ovarian function (23, 24).

Additionally, two patients in Ferro et al. study underwent both oocyte and embryo cryopreservation simultaneously (28).

### 3.7 Pregnancy and reproductive outcomes

Although fertility preservation was pursued by cancer patients in all seven studies, most participants did not return to use their stored reproductive material (e.g., cryopreserved oocytes, embryos, or ovarian tissue) during the study follow-up period. In Silva et al., none of the breast cancer patients who cryopreserved oocytes attempted pregnancy using their stored gametes (22). Similarly, Cobo et al. reported that many patients who had undergone fertility preservation had not yet pursued embryo transfer (25).

Among those cancer patients who did attempt pregnancy using their cryopreserved specimens, success rates varied. Cobo et al. reported a baseline AMH level of 2.16 ng/ml and clinical pregnancy rates of 41.4% for fresh embryo transfers and 32.1% for frozen transfers among onco-fertility patients, yielding a cumulative live birth rate of 35.2% (25). Similarly, Dolinko et al. documented baseline AMH levels of 2.8 ng/ml, with a 40% live birth rate per cycle and a 42.1% success rate per embryo transfer among 15 of the 147 cancer patients who proceeded with frozen embryo transfers (27).

Notably, spontaneous pregnancies were more common than those achieved through assisted reproductive technologies (23, 24, 28). Ferro et al. reported 11 pregnancies, 10 of which occurred spontaneously (28), while Ciccarone et al., 2023 documented nine pregnancies, all conceived naturally, without the use of cryopreserved material (24).

## 4 Discussion

We conducted a systematic review of the existing evidence on AMH screening in female cancer survivors before, during, and after chemotherapy, focusing on those wanting to pursue fertility preservation. We sought to identify gaps in the literature regarding AMH as a prognostic indicator of reproductive outcomes, highlighting areas that require further investigation. By synthesizing the available data, we aspired to: i) inform future research on AMH screening and fertility preservation, and ii) contribute to the development of data-driven recommendations for clinical practice.

Our analysis revealed that AMH levels were highest in healthy controls compared to those with local and systemic cancers (26), and among breast cancer patients prior to chemotherapy compared to during and after chemotherapy (at 1 month and at last available follow-up) (22) (**Table 4**). In contrast, cohorts predominantly with breast cancer and hematologic cancer patients had higher AMH levels at baseline compared to controls (**Table 4**) (25, 26). Only one study (22) assessed AMH levels during chemotherapy, reporting a dramatic AMH decline in breast cancer patients.

Following chemotherapy, AMH levels remained consistently low across all four studies that reported post-treatment values. However, the degree of AMH recovery varied, influenced by cancer type, treatment regimen, and patient age.

### 4.1 In the future, could AMH potentially serve as a reliable and valid biomarker of pregnancy and live birth in women with cancer who undergo fertility preservation

Anti-Müllerian hormone (AMH) is widely recognized as a marker of ovarian reserve, but its predictive value for reproductive success, particularly pregnancy and live birth in cancer patients undergoing fertility preservation, remains uncertain. While AMH has been useful in assessing ovarian reserve, its role as a predictor of pregnancy and live births remains unclear in the context of cancer patients undergoing fertility preservation. High AMH levels prior to chemotherapy permit cancer patients to cryopreserve more oocytes/embryos, thereby increasing their chances of successful subsequent pregnancy outcomes using preserved gametes/embryos.

Only three studies (25–27) provided relevant data on cancer patients who returned to use their cryopreserved specimens post-chemotherapy, offering limited but valuable insights into pregnancy and live birth outcomes (**Table 2**). Cobo et al. (25) reported baseline AMH levels of 2.2 ng/ml, along with higher clinical pregnancy rates following fresh embryo transfers, yielding a cumulative live birth rate of 35%. Similarly, Dolinko et al. (27) documented baseline AMH levels of 2.8 ng/ml with a 40% live birth rate per cycle (**Table 2**) among 15 patients who utilized frozen embryos. Nurudeen et al. recorded baseline AMH levels of 1.3 ng/ml and two pregnancies from those who used preserved specimens, one of which resulted in a live birth, while the other was still pregnant at the study conclusion (25).

Hence, these findings suggest that baseline AMH levels may serve as a valuable reference for women considering fertility preservation. However, it is important to note that these three studies provided only baseline AMH data, with no information on post-treatment levels. More significantly, none of these studies examined nor linked baseline AMH levels with fertility preservation birth outcomes in individual cancer patients, which was a central focus of our investigation. In summary, high AMH levels prior to chemotherapy may permit cancer patients to cryopreserve more oocytes/embryos, thereby potentially increasing their chances of successful pregnancy outcomes using preserved gametes/embryos.

Interestingly, spontaneous pregnancies were more commonly reported in all studies than those achieved through assisted reproductive technologies, likely due to limited follow-up. Of note, the total number of women attempting to achieve pregnancy post-chemotherapy were not reported in these studies. Nevertheless, Ferro et al. (28) reported 10 natural pregnancies out of eleven, while Ciccarone et al., 2023 (24) documented all nine pregnancies as spontaneous. Additionally, Ciccarone et al., 2020 (23) recorded all seven pregnancies as spontaneous. However, it remains unclear from these three studies, as well as Silva et al. (22), whether these cancer patients/survivors returned to use their stored specimens or failed to conceive naturally during the study period.

Our findings encapsulate the current state of knowledge of AMH and fertility preservation outcomes in cancer survivors and will pave the way for future advancements in the field. The limited number of studies, coupled with the variability of follow-up periods, hindered the comprehensive assessment of post-treatment AMH recovery. As a result, definitive conclusions regarding the optimal time frame for utilizing stored reproductive specimens or the period when AMH levels tend to recover most robustly remain elusive.

These findings highlight the need for further research to establish whether AMH, beyond its role as an ovarian reserve marker, can serve as a clinically meaningful predictor of successful conception and live birth in cancer survivors using their specimens for fertility preservation. Future studies should focus on standardized AMH measurement methods, longitudinal studies tracking AMH recovery post-chemotherapy, and larger, more diverse patient cohorts to strengthen the evidence base.

### 4.2 Study details that prevented us from advancing knowledge in the field

#### 4.2.1 AMH measures

Determining the role of AMH in predicting fertility preservation success is exceedingly challenging due to a host of study limitations. Variable study designs (e.g., retrospective, prospective, randomized clinical trials) performed across different countries complicate direct comparisons. This inconsistency makes it difficult to interpret whether AMH measurements prior to treatment can reliably reflect a woman’s reproductive potential or fertility preservation outcomes. Additionally, AMH levels may be influenced by individual factors such as body mass index (29, 30), BRCA gene status (31–35), as well as cancer treatment characteristics (36–39), complicating interpretation. Additional factors including AMH measurement techniques, patient’s age, and cancer types were summarized in this systematic review (**Tables 2**, **4**).

The use of a single AMH assay during and after chemotherapy for cancer treatment provides limited insight into ovarian reserve. Since AMH levels fluctuate over time and are influenced by variations in measurement techniques, relying on a single measurement offers incomplete insight. Furthermore, studies that tracked women to conception measured AMH only once without monitoring its changes over time, which limits the ability to understand the full impact on fertility.

#### 4.2.2 AMH measurement techniques

AMH measurement techniques have evolved, but earlier assays lacked sufficient sensitivity to detect very low AMH levels, further limiting their utility. More recently, automated assays from Beckman Coulter and Roche Diagnostics have improved precision and sensitivity. However, significant variations in laboratory techniques still exist. For example, assays like ELISA produce different results compared to the Gen2 Beckman manual method, resulting in inter- and intra-assay discrepancies. Some assays, such as those from Ansh Laboratories, have yielded even higher AMH values, further complicating interpretation (40).

Beyond assay variation, differences in sample storage and stability may further contribute to inconsistent findings. The absence of assay standardization raises concerns about reliability and reproducibility, highlighting the need for an international consensus to improve AMH measurement accuracy and interpretation (41).

#### 4.2.3 Clinical factors affecting AMH

Further complexity of interpreting AMH as a prognostic marker arises from the diverse fertility preservation methods (e.g., cryopreserved embryos, oocytes, and tissues), cancer types, and chemotherapy regimens, all of which impact AMH levels and reproductive outcomes. Additionally, variability in patient demographics, including age, ethnicity, and cancer diagnosis, further complicate the consistent interpretation of AMH as a prognostic marker of fertility.

The administration of a GnRH analogs (GnRHa) during chemotherapy to reduce ovarian failure and increase pregnancy rate is well documented. Nevertheless, the available data on AMH behavior during concurrent administration of chemotherapy and GnRHa administration are inconsistent, adding another layer of complexity (42).

A critical limitation in many studies is the lack of clarity regarding the utilization of stored specimens during fertility preservation. Many studies fail to specify whether women attempted pregnancy using their preserved specimens or conceived naturally. Additionally, the number of patients returning to use their fertility preservation specimens was exceedingly low, making it challenging to draw robust conclusions about the effectiveness of fertility preservation strategies in cancer survivors.

### 4.3 Systematic review limitations

Many studies in this systematic review had small sample sizes (e.g., n=46), with cancer survivor groups ranging from 46 to 1073.

Cancer diagnoses spanned multiple types and stages, yet these details were often not reported, limiting the ability to assess their impact. Additionally, treatment specifics, including type, duration, cumulative drug dose, radiation dose, and targeted location, were rarely disclosed.

Control groups included both healthy women and cancer patients who did not undergo the same treatments, introducing potential bias. Furthermore, the follow-up durations for cancer survivors varied considerably, making it difficult to assess long-term reproductive outcomes. The timeframes ranged from 1 month to 36 months following chemotherapy (**Table 4**), mirroring the clinical uncertainty about the optimal follow-up period needed to observe successful healthy live births.

Most studies primarily examined ovarian reserve rather than clinically meaningful endpoints such as pregnancy or live birth. This likely reflects the difficulty in recruiting a sufficient number of cancer patients who return to use their cryopreserved specimens and complete fertility preservation. Statistical measures (medians vs. means) further hindered AMH comparisons across studies.

Potential bias could exist in the interpretation of pregnancy rates due to associated co-morbidities and male infertility. Additionally, confounding factors were inconsistently accounted for, and lifestyle influences such as smoking, alcohol use, recreational drugs, caffeine intake, psychological stress, physical activity, vitamin D levels, and obesity were largely overlooked. Many studies also lacked details on medical and reproductive histories, as well as prior hormone use. These gaps further limited the ability to draw definitive conclusions about AMH’s predictive value for fertility preservation outcomes.

The limited number of eligible studies (n=7) reflects the nascence of this research rather than shortcomings in our process. The novelty of our hypothesis of measuring AMH levels before, during, and after chemotherapy in women who elected fertility preservation meant working with a small and heterogeneous evidence base. Each of the studies contributed important but partial insights, often focusing on one or two discrete time points (e.g., before or after chemotherapy).

As a result of these deficiencies, the current body of evidence remains insufficient to draw definitive conclusions about whether AMH can reliably predict fertility outcomes in cancer survivors choosing fertility preservation. The limitations are not a reflection of the quality of our review, but rather a reflection of the early stage of development (infancy) of this important and complex field.

### 4.4 Systematic review advantages

To date, this systematic review is the first to comprehensively identify, synthesize, and evaluate all available evidence on AMH and fertility preservation outcomes in cancer survivors. Beyond consolidating existing knowledge, our analysis also highlighted critical gaps in the literature, underscoring areas that require further investigation. By employing a precise and transparent methodology, we minimized bias and strengthened the reliability of our findings.

The clinical relevance of our research question further strengthens the significance and credibility of this review. Understanding which women are most likely to achieve successful fertility preservation outcomes whether through the use of cryopreserved oocytes/embryos or through natural conception after completing cancer treatment, is essential for the growing population of reproductive-aged cancer patients seeking to preserve their future fertility. Our systematic review provides the backbone for future research by identifying key exposures, outcomes, and potential confounders that should be considered to answer this important question.

### 4.5 Conclusions and future recommendations

Further research is needed to define optimal AMH levels before, during, and after cancer treatment as well as to understand the timeline for AMH restoration and its implications for fertility preservation outcomes. Future studies should identify optimal AMH levels in conjunction with such factors as age, type of cancer, and treatment regimens, along with the ideal post-chemotherapy AMH timeframe (e.g., 24 or 36 months) for achieving a healthy live birth following fertility preservation.

To improve the quality of future studies, it is essential to standardize AMH measurement techniques, coordinate the timing of assessments, and focus on similar cancer types. Separating natural and assisted reproductive outcomes will also enhance the reliability of findings. These improvements will offer valuable insights to inform critical decisions for female cancer patients, clinicians, and policymakers, ultimately helping to optimize strategies and outcomes of fertility preservation–a life-transforming option for many cancer survivors.
